# Quality improvement in remote prescribing

**DOI:** 10.1192/bjo.2021.474

**Published:** 2021-06-18

**Authors:** Mohamed Bader, Ibtisam Abbas, Joanna Peacock

**Affiliations:** 1 Royal Gwent Hospital; 2 ysbyty ystrad fawr; 3 neville hall hospital

## Abstract

**Aims:**

To evaluate attitudes in prescribing and utilising 'As Required' (referred to as PRN/Pro Re Nata) sedating medications (Benzodiazepines, Z-Drugs, Anti-psychotics, and Promethazine)

To evaulate current remote prescribing processes and improve safety and transparency

**Method:**

Plan:

Review of remote prescribing policy. It was highlighted that current practice was not in line with NMC guidance of the time as no follow-up written instruction by a doctor was received. Concerns were also raised about the general safety of verbal communication of prescriptions out of hours. A survey was conducted to assess attitudes towards the prescription of ‘PRN medication’ and the role of psychological therapies as an alternative to both doctors and nurses working in ABUHB's Mental Health and Learning Disabilities division.

Do:

Survey results showed a nuanced response from both doctors and nurses but an agreement that there is a role for as required medication, especially in the context of acute mental distress, indicating safety around the process rather than elimination/reduction of PRN medication prescribing would be desired. This lead to an overhaul of the out of hours prescribing process between junior doctors and those receiving the ‘verbal order’ as detailed below: Phone conversation between a junior doctor and ward nurse receiving the verbal order. A digital form is then completed by the ward nurse including current regular medication, PRN medication (including times of use), physical health history, and any additional requested information such as QTc on 12 lead electrocardiogram (ECG) or current vital signs. The junior doctor may assist with obtaining the relevant information but there are clear prompts on the form, to ensure the pertinent questions regarding safe prescribing are considered by both parties. The dose and route of the medication are clearly documented by the junior doctor as well as time of prescription and the form is emailed back to the ward nurse. This process is far more transparent and much less prone to errors due to miscommunication. a. The prompts also save time ensuring the relevant information is on hand prior to discussion as opposed to searching for medication charts, ECGs, etc. b. Highlighting the importance of QTc monitoring to encourage safe prescription of anti-psychotics and Promethazine c. The prompts also highlight the importance of physical health and current vital signs with regards to safe prescribing d. The prompts are stored on a network drive alongside other verbal orders allowing for easier future auditing off remotely off and on site These changes were highlighted via email, junior doctor forums, and induction of new doctors.

Study

A Round 2 survey was drafted to evaluate the new process and forms with an aim to ensure uptake and to identify any issues. Despite using the same channels to identify survey participants, the response rate was much lower than the Round 1 survey. See Round 2 results.

Act

With the limited feedback obtained the main issue identified was with regards to rapid tranquilisation of an aggressive patient who poses a risk to self and others. In this scenario it was deemed a risk to wait for an email form to be completed. Clarification emails were sent to relevant professionals to clarify that the rapid tranquilisation policy does allow for verbal orders with a subsequent digital order form to be completed at a later time when it is safe to do so.

**Result:**

Round 1

- Nurses n = 26
- Doctors n = 27
- Nursing
- 92% routinely request Z-Drugs and Benzodiazepines for treatment of insomnia
- 88% routinely request Benzodiazepines for treatment of agitation
- 73% routinely request Promethazine for for treatment of agitation
- 69% routinely request PRN Anti-Psychotics for treatment of agitation
- 35% would routinely request Promethazine for treatment of insomnia
- 19% would routinely request Haloperidol without a recent ECG (>3 months)
- 15% would request Benzodiazepines for treatment of psychotic symptoms
- 12% would request Lorazepam above British National Formulary maximum doses
- As required medications dispensed per shift
- 54% report 0 to 3 times
- 23% report 4 to 6 times
- 23% report 6 to 10 times
- Agitation was most commonly defined as
- 96% hostile behaviour/physical aggression
- 92% hostile/threatening/derogatory speech
- 81% visible anxiety
- 69% disturbed behaviour that is not threatening/derogatory towards others
- 31% patient reported anxiety without objective evidence
- PRN medication use reviewed by doctors
- Daily (8%)
- Weekly (85%)
- Monthly (8%)
- 5 most commmonly cited reasons contributing to PRN medication use
- 77% Ward atmosphere (ie. volatile ward environment)
- 69% Patient depdence (psychological/physiological)
- 54% Patient expectation
- 42% Limitted expectation of benefit from psychological skill utilisation
- 42% Usual habit/culture of prescribing by doctors
- What are your thoughts on the use of psychological interventions in an acute setting? [Open Ended, n = 22]

Reviewing the themes from the open ended responses:

- Nursing staff feel positively about psychological interventions in the right setting at the right time but find challenges to delivering them. Some staff cite the fact that a patient is admitted indicates their level of acuity requiring PRN utilization. Some responses indicate that patients may be medicating the normal human experience. Ward atmosphere, how ill the patient currently is, patient willingness, staff shortages, paperwork taking priority, lack of training in psychological therapies were all cited as challenges.
- Doctors
- 96% routinely prescribe Benzodiazepines for treatment of agitation
- 92% routinely prescribe Z-drugs and Benzodiazepines for treatment of insomnia
- 63% routinely prescribe PRN Anti-psychotics for treatment of agitation
- 38% routinely prescribe Promethazine for treatment of agitation
- 29% routinely prescribe Promethazine for treatment of insomnia
- 25% routinely prescribe Benzodiazepines for treatment of psychosis
- 12.5% routinely prescribe Lorazepam above British National Formulary maximum doses
- 8% routinely prescribe Haloperidol without a recent ECG (>3 months)
- Rapid Tranquilisation Policy
- 70% of doctors were familiar with the up to date Rapid Tranquilistion Policy
- 5 most commmonly cited reasons contributing to PRN medication use
- 19% nursing staff shortages
- 15% ward atmosphere (ie. volatile ward environment)
- 15% nursing staff expectations
- 11% usual habit of prescribing
- 11% patient expectations
- What are your thoughts on the use of psychological interventions in an acute setting (n = 26)?

Reviewing the themes from the open ended responses:

- Doctors are somewhat divided in their approach to psychological approaches, the majority stating or alluding to it being a first line management option but some citing staffing levels to be a deterrent. Others had a more nuanced view of it rather than a general first line treatment, requiring risk/benefit analyses before use. The minority did not know enough about psychological interventions or thought it often doesn't work.
- Round 2
- Nurses n = 8
- Doctors n = 8
- Nursing
- Total responded n = 8
- Acute psychiatric ward nurses n = 4
- Psychiatric intensive care unit nurses n = 4
- 50% were unaware that physical health emergencies and rapiq tranquilisation can allow for the older process of 'verbal orders' followed by the form due to the imminent risks associated with delaying treatment to complete the form
- 100% (n = 8) were familiar with the digital order forms
- 87.5% (n = 7) were familiar with the digital order policy
- With regards to form locations
- 87.5% (n = 7) had access to blank forms and would store them alongside paper medication charts
- 12.5% (n = 1) were not aware that the ‘verbal order’ policy was not digitised

With regards to digitised order requests:

- 75% (n = 6) did not report any change the frequency of requesting out of hours prescriptions
- 12.5% (n = 1) reported a reduction in requests
- 12.5% (n = 1) reported an increase in requests
- 75% (n = 6) reported that the digital order form puts up barriers to requesting medication out of hours

With regards to the form:

- 12.5% (n = 1) report that the form helps them formulate their requests
- 50% (n = 4) report that the form requires the appropriate amount of information
- 12.5% (n = 1) report that the form requires too much information
- 37.5% (n = 3) did not comment on the amount of information the form requires

With regards to safety:

- 25% (n = 2) report that the digitised system is safer
- 75% (n = 6) did not comment on safety

With regards to time to fill out the form:

- 87.5% (n = 7) report that the form is more time consuming
- 12.5% (n = 1) did not comment on time consumption

If given the option to revert to verbal orders:

- 37.5% (n = 3) would like to revert back to the old system
- 25% (n = 2) would like to remain on current system
- 37.5% (n = 3) did not comment on which system they'd prefer
- Doctors
- Total responded n = 8.
- Consultants n = 2
- Staff Grade doctors n = 1
- Core Trainees in Psychiatry n = 3
- Fixed term appointees n = 2
- 100% (n = 8) were familiar with the up to date rapid tranquilisation policy
- With regards to the digital order forms
- 62.5% regularly see them in patient files (n = 5)
- 37.5% occasionally become aware of them (n = 3)
- 0% were unaware of the new digital order forms (n = 0)
- With regards to inappropriate out of hours prescriptions
- 37.5% report that there was a reduction (n = 3)
- 50% report there being no significant change (n = 4)
- 12.5% report there being an increase (n = 1)

With regards to safety:

- n = 6 reported the new system to be safer
- n = 2 did not comment on safety

With regards to time:

- n = 2 report it being more time consuming to use the digital orders
- n = 6 did not comment on time consumption
- With regards to returning to verbal order forms
- n = 3 would like to remain on digital orders
- n = 5 did not comment on returning to verbal order forms

Other:

- n = 2 commented in the comment box that this change was overdue
- n = 1 commented that the forms give insight into patient presentations and management

**Conclusion:**

Doctors routinely prescribe Z-drugs and benzodiazepines, and would generally consider Haloperidol as a second line over Promethazine (while nurses had a slight preference for requesting Promethazine over Haloperidol). The role of 12 lead electro-cardiogram monitoring would require further exploration in separate audits, as both Promethazine and Haloperidol can cause QTc interval prolongation [4,5].

Doctors most commonly cited expectations by nursing staff as the main driver for PRN medication prescription. Profound differences were present with regards to rationale behind PRN medication use when comparisons between doctors and nurses self-reports were made. The majority of nurses cited ward atmosphere and patient dependence/expectation as main drivers, whereas a minority of doctors shared those views. This represents a concerning disconnect between professionals, although it can be explained by the higher proportion of time ward nurses spend on mental health wards and in direct patient care. Nursing staff, being the dispensers of medication, would also likely be the main professionals contacted for the request of PRN medication by patients.

Nuanced views were given to the role of psychological redirection. This was shared between doctors and nurses, although many cited concerns about nursing staff shortages leading to a possible overreliance on PRN medication. A minority of doctors (n = 2) would recommend psychological redirection after first line rapid tranquilisation was exhausted. The counterargument being that someone admitted onto a ward tacitly implies a high level of acuity and reduced appropriateness of psychological techniques.

Hypnotics most commonly being requested likely reflects the difficult nature to initiate and maintain sleep is an acute ward setting.

On review of the Round 2 results indicate that doctors and nurses agree that the new system is safer although more time consuming. Concerns were raised about rapid tranquilisation and immediate emergencies, although the revised policy would allow for the verbal order policy to be followed with a digital order in these circumstances. This was clarified via further communication with relevant parties.

The changes were more received more positively by doctors than nurses, with some nurses opting for the older system if possible. It was also raised that this may be putting up barriers for out of hours prescriptions, although the required information is arguably succinct and only requests vital information for safe prescribing. Further exploration of these concerns would be indicated. The Round 2 results were limited by the low sample size compared to the first round.

Despite the limitations and concerns about the new system, digitising the system allows for further audits and studies to utilize much more robust methods of measuring out of hours prescriptions than self-reported measures employed in the initial rounds. Although they may not be directly compared to findings of this report, future baselines can be established and compared to in an objective manner.

Future Rounds

Proposed: To design and clearly display information on commonly requested medication by patients, empowering them to make more informed decisions on the medications they request. This could be in the form of leaflets patients could take or posters on areas where patients receive medication. One example is that Zopiclone is a very commonly requested medication on an as required basis although patients may not be as aware of the risks associated with chronic use.

Proposed: To design and clearly display information on psychologically informed techniques in patient areas such distress tolerance and sleep hygiene. This would be on mental health sites which do not currently display this information. To measure impact on PRN medication dispensation.

Proposed: Further exploration of patient perceived ward environment and measures that can be implemented to reduce anxiety/insomnia associated with inpatient admission.

Proposed: Exploration of proportion of inpatient initiated PRN medication progresses to long term use in the community (largely focused on hypnotics and benzodiazepines).

